# Indikation zur Systemtherapie bei Psoriasis: Kommentierte Checkliste für die Praxis

**DOI:** 10.1111/ddg.15613_g

**Published:** 2025-02-06

**Authors:** Ralph von Kiedrowski, Ulrich Mrowietz, Matthias Augustin

Die Systemtherapie der mittelschweren bis schweren Psoriasis hat sich zu einem der wichtigsten Pfeiler in der Versorgung entwickelt und weist eine wachsende Vielfalt auf.^1^, ^2^, ^3^ Für eine qualitätsgesicherte dermatologische Arzneimittelversorgung sind Standards in der Indikationsstellung von hoher Wichtigkeit. Dies gilt insbesondere für den Einsatz therapeutischer Innovationen. Eine essenzielle Anforderung an entsprechende Standards ist deren hohe Verständlichkeit, Eindeutigkeit, rechtliche Sicherheit und praktische Handhabbarkeit. Die Praktikabilität ist von besonderer Bedeutung. Für die Indikationen atopische Dermatitis, Vitiligo und chronische Prurigo wurden entsprechende Checklisten bereits entwickelt und in den Leitliniengruppen konsentiert.^4^, ^5^, ^6^

**ABBILDUNG 1:**
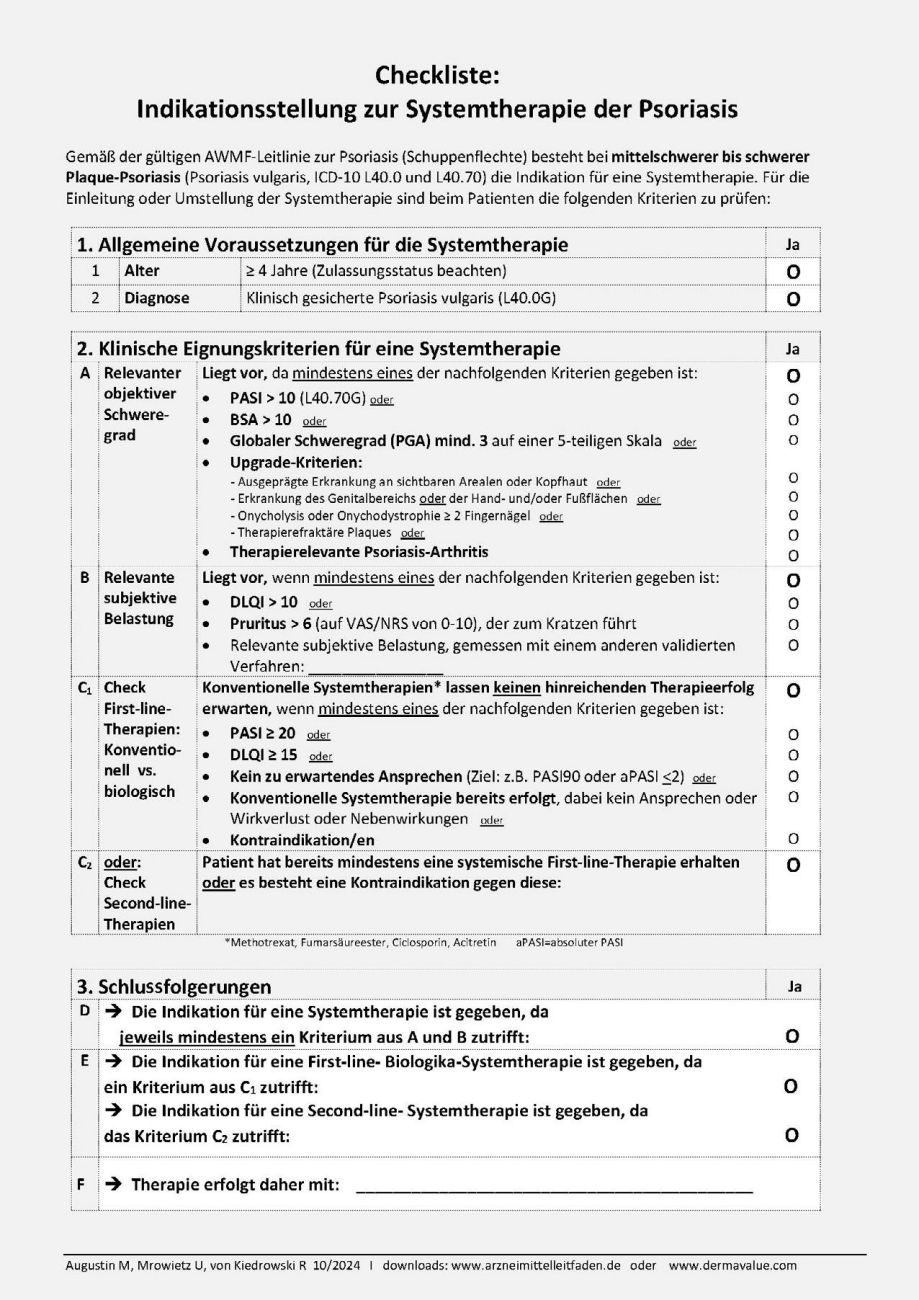
Checkliste zur Indikationsstellung der Systemtherapie bei Psoriasis

Von Mitgliedern der S3‐Leitliniengruppe zur Psoriasis wurde jetzt auch eine Checkliste zur Indikationsstellung der Systemtherapie bei Psoriasis entwickelt (Abbildung 1), die sich inhaltlich an die geltende S3‐Leitlinie anlehnt.

Dem grundsätzlichen Aufbau aller Checklisten folgend, wurden dabei Kriterien zur Ermittlung des objektiven Schweregrades (A), der subjektiven Krankheitsbelastung (B) und der vorausgehenden Therapien (C) definiert. In der finalen Fassung wurden fünf alternative Kriterien eines hinreichenden objektiven Schweregrades, sowie sechs Kriterien eines subjektiven Schweregrades konsentiert. In beiden Bereichen besteht zudem die Möglichkeit des begründeten Einsatzes weiterer Messparameter, sodass auch besondere Therapiesituationen berücksichtigt werden können. Im dritten Teil wird wie in den Checklisten für die anderen Indikationen sichergestellt, dass vorausgehende Therapien vor Einsatz einer Systemtherapie erwogen, nicht aber zwingend angewendet werden müssen. Der letztere Sachverhalt ist dann gegeben, wenn keine ausreichende Aussicht auf Erfolg einer topischen oder UV‐Therapie besteht oder gegen diese eine oder mehrere Kontraindikationen bestehen. Die Kriterien im Detail:

- Ein relevanter objektiver Schweregrad liegt vor, wenn mindestens eines der nachfolgenden Kriterien gegeben ist: Psoriasis Area and Severity Index (PASI) > 10, Body Surface Area (BSA) > 10, Physician Global Assessment (PGA) mindestens 3 auf einer 5‐teiligen Skala. Alternativ kann auch ein Kriterium (ein sogenanntes „Upgrade‐Kriterium“) aus dem europäischen Expertenkonsensus vorliegen.^7^
- Eine relevante subjektive Belastung liegt vor bei Dermatology Life Quality Index (DLQI) > 10 oder Pruritus > 6 (auf Visual Analog Scale (VAS) / Numeric Rating Scale (NRS) von 0–10), besonders, wenn dieser zum Kratzen führt. Wichtig ist auch, dass für den relevanten subjektiven Schweregrad („Krankheitslast“) noch weitere Kriterien herangezogen werden können, wenn diese für den einzelnen Patienten in Zusammenhang mit der Psoriasis von Bedeutung sind, zum Beispiel klinisch relevante Depression, Angst oder Wohlbefinden (Tabelle 1). Diese sind mit validierten Instrumenten zu messen.
- Wie in den vorausgehenden Checklisten ist zunächst zu prüfen, inwieweit die individuellen Therapieziele auch mit einer topischen oder UV‐Therapie erreicht werden könnten. Ist dies nicht der Fall, ist eine Systemtherapie indiziert. Hier gilt es bei Psoriasis die Kriterien für eine Erstlinien‐ (First‐Line‐Therapy) oder Zweitlinientherapie (Second‐Line‐Therapy) zu prüfen. Im Bereich der Erstlinientherapie sind grundsätzlich zunächst die konventionellen Systemtherapien (zurzeit sind dies Fumarsäureester, Methotrexat (MTX), in Ausnahmefällen Ciclosporin und Acitretin) zu prüfen. Ein Einsatz von Biologika in der Erstlinientherapie ist indiziert, wenn kein hinreichender Therapieerfolg mit den konventionellen Systemtherapeutika zu erwarten ist. Hierzu zählen folgende alternative Kriterien: a) Bei Indikationsstellung ist der Schweregrad PASI ≥ 20 oder DLQI ≥ 15; b) kein zu erwartendes Ansprechen (z. B. PASI 90 oder ein absoluter PASI (aPASI) <2) auf Nicht‐Biologika; c) konventionelle Systemtherapie bereits erfolgt, dabei kein Ansprechen oder Wirkverlust; d) Erwartung oder Beobachtung von Nebenwirkungen oder Kontraindikation/en gegen konventionelle Systemtherapien. Bei b) kommen in besonderen Therapiesituationen spezifische Therapieziele in Betracht, etwa bei Nagelpsoriasis oder schwere Kopfhaut‐Psoriasis. Eine Zweitlinientherapie kann indiziert sein, wenn der Patient bereits mindestens eine systemische Erstlinientherapie erhalten oder es Kontraindikation gegen die Erstlinientherapien gibt.

**TABELLE 1:** Beispiele für weitere relevante Parameter zur Erfassung der Krankheitslast, die im Einzelfall in der Checkliste unter (B) verwendet werden können.

Parameter	Messinstrument	Anzahl Items	Skala von‐bis	Cut‐off für hohen Schweregrad
Depression	HADS‐D	7	0–42^#^	>15
Angst	HADS‐A	7	0–42^#^	>15
Depression, Screening	PHQ‐2	2	0–6^#^	>3
Angst, Screening	GAD‐2	2	0–6^#^	>3
Psychische Gesundheit, Wohlbefinden/Wellbeing	WHO‐5	5	0–25^+^	<13
Gesundheitsstatus, visuell‐analoge Skala	EQ‐VAS	1	0–100^+^	<80
Gesundheitsstatus	EQ‐5D‐5L	5	0–25^+^	<20

HADS‐D, Hospital Anxiety and Depression Scale; HADS‐A, Hospital Anxiety and Depression Scale‐Anxiety subscale; PHQ‐2, Patient Health Questionnaire‐2; GAD‐2, Generalized Anxiety Disorder; WHO‐5, WHO‐Five Well‐Being Index; EQ‐VAS, European Quality of Life Visual Analogue Scale; EQ‐5D‐5L, European Quality of Life 5 Dimensions 5 Level Version; ^+^Hoher Wert entspricht geringer Belastung; ^#^Hoher Wert entspricht hoher Belastung.

Mit der vorliegenden Checkliste wurde eine Lücke in der Versorgung der Psoriasis mit systemischen Arzneimitteln geschlossen, indem reproduzierbare Kriterien für die Indikationsstellung zur Systemtherapie und deren differenzielle Auswahl definiert wurden. Die Kriterien stimmen grundsätzlich mit der aktuellen Fassung der S3‐Leitlinie zur Therapie der Psoriasis überein.^8^ Die Anwendung dieser praxisnahen Checkliste wird für die dermatologische Versorgung in Deutschland empfohlen. Wünschenswert ist eine nachfolgende Implementierungsforschung, mit der der Nutzen dieser Checkliste systematisch geprüft und die gewählten Kriterien gegebenenfalls adaptiert werden. Letzteres wird auch dann zu erwägen sein, wenn die erwartete Neuauflage der Konsensus‐Kriterien für den Schweregrad der Psoriasis und die daraus abgeleiteten Therapieziele publiziert sind.
